# Drainage of middle lobe vein into anomalous right lower lobe vein: management during thoracoscopic lobectomy

**DOI:** 10.1002/rcr2.568

**Published:** 2020-05-05

**Authors:** Dario Amore, Alessandro Saglia, Dino Casazza, Tullio Valente, Pasquale Imitazione, Carlo Curcio

**Affiliations:** ^1^ Department of Thoracic Surgery Monaldi Hospital Naples Italy; ^2^ Department of Respiratory Diseases Monaldi Hospital Naples Italy; ^3^ Department of Radiology Monaldi Hospital Naples Italy

**Keywords:** Lung cancer, vascular anomalies, VATS lobectomy

## Abstract

Variations in pulmonary venous anatomy should not be underestimated by thoracic surgeons prior to or during major lung resections in order to avoid serious surgical complications. Here, we report a case of middle lobe vein draining into a right inferior lobar vein formed by two anomalous trunks lying on the superior surface of the common basal bronchus: in such instance, to avoid compromising the middle lobe vein drainage during a thoracoscopic right lower lobectomy, the two main tributaries of the lower lobe vein were individually identified and dissected peripherally from the anterior aspect after division of the major fissure. A careful hilar dissection and a precise surgical strategy can help surgeons perform correct procedures in presence of pulmonary vascular anomalies.

## Graphical abstract

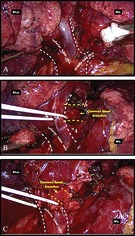

## Introduction

The current scientific literature documents several imaging findings and systematic overview of pulmonary vein abnormalities provided by radiologists for a correct recognition of aberrant vessels and a more precise identification of pulmonary nodules or intersegmental lymph nodes near to vascular structures. In the last decade, also in other fields such as the thoracic surgery, many authors have reported a wide spectrum of venous pulmonary anomalies identified preoperatively or intraoperatively in order to make lung major resections safer during video‐assisted thoracoscopic surgery (VATS) where a short exposition of the pulmonary hilum can lead to mistakes due to incorrect identification of anatomical variations [1, 2, 3]. This report shows that, in some instances, pulmonary vascular anomalies require not only a meticulous hilar dissection, but also a different surgical strategy to achieve safe surgical resections.

## Case Report

**Figure 1:**
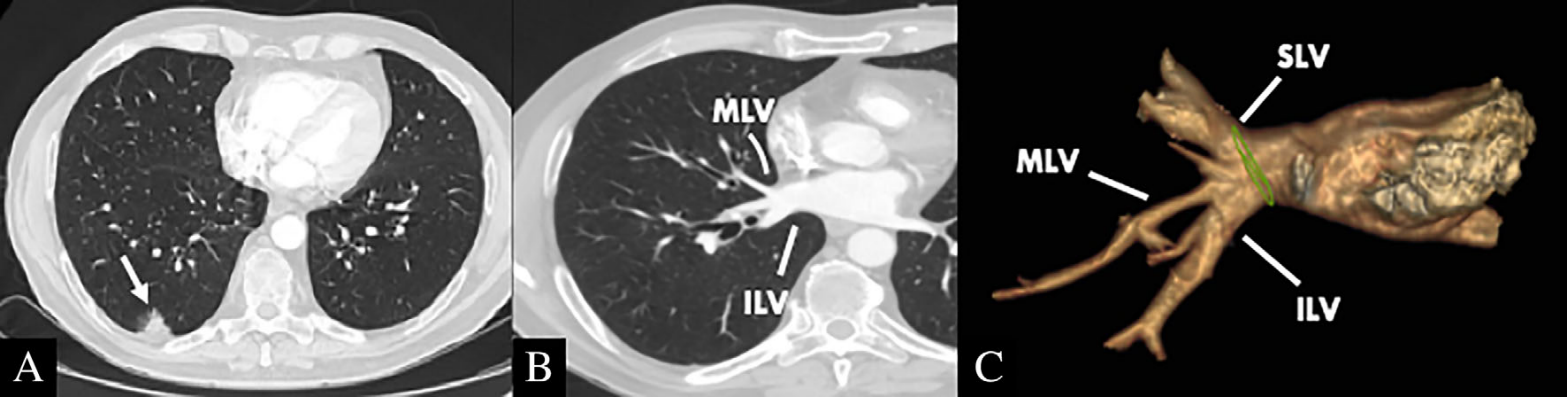
Preoperative enhanced chest CT scans. (A) NSCLC located in the right lower lobe (white arrow). (B) MLV joins the ILV to form the right inferior pulmonary vein. (C) 3D CT reconstruction of right pulmonary veins shows the MLV and ILV forming a single venous confluence. 3D, three‐dimensional; CT, computed tomography; ILV, inferior lobar vein; MLV, middle lobe vein; NSCLC, non‐small cell lung cancer; SLV, superior lobar vein.

**Figure 2:**
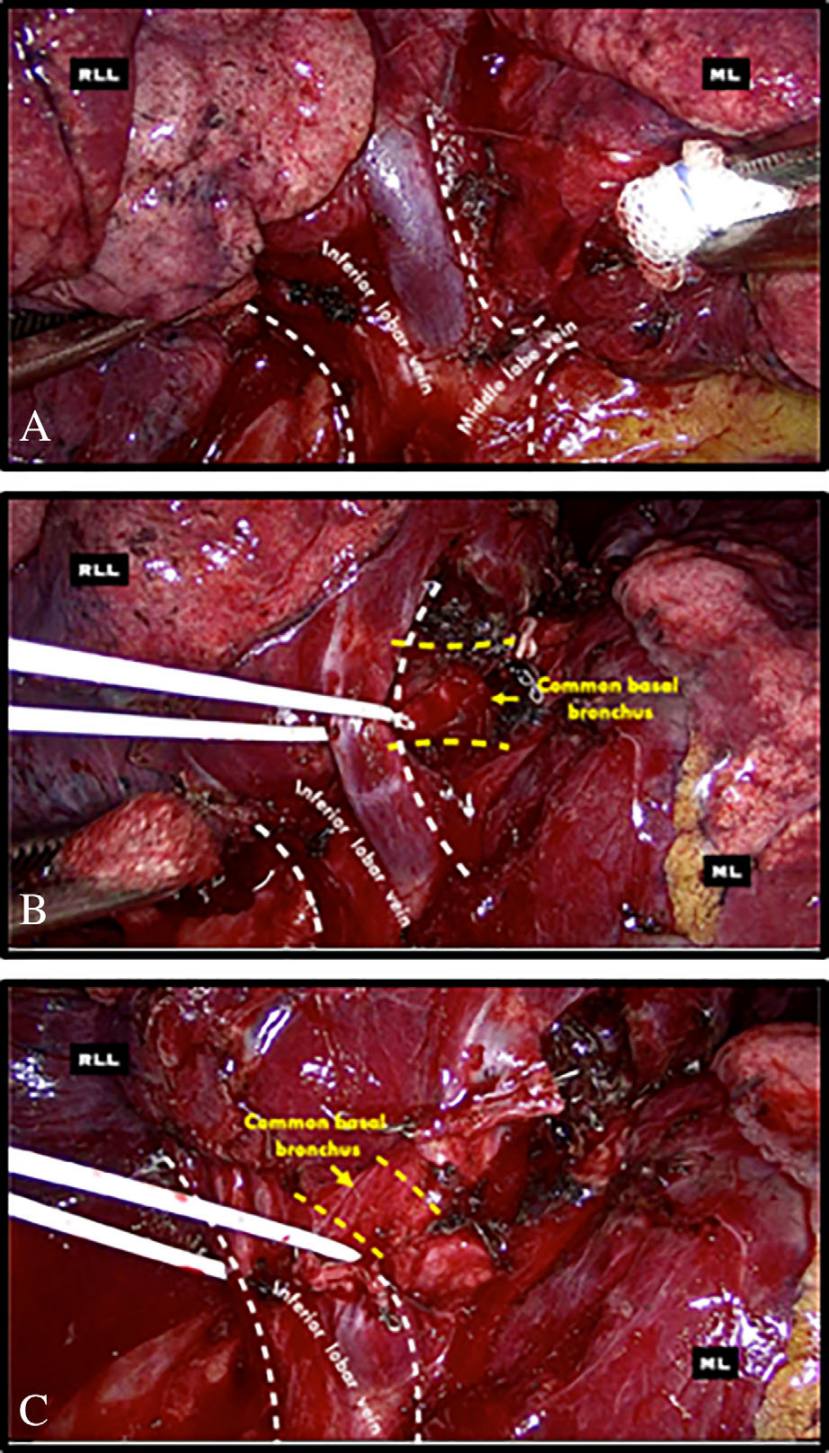
Intraoperative view of the right pulmonary hilum. (A) Inferior pulmonary vein draining the RLL and ML. (B, C) Inferior lobar vein composed of two main trunks lying on the superior surface of the common basal bronchus, dissected from the anterior aspect, and individually encircled with a vessel loop. ML, middle lobe; RLL, right lower lobe.

A 67‐year‐old man was admitted to our unit for treatment of non‐small cell lung cancer (NSCLC). Preoperative chest computed tomography (CT) scans showed a pseudo‐nodular thickening in the right lower lobe (Fig. 1A) and a vascular anomaly formed by the confluence of the middle lobe vein and inferior lobar vein (Fig. 1B, C). Positron emission tomography (PET) scan demonstrated focal fluorodeoxyglucose uptake in the right lower lobe; the standardized uptake value of the histologically proven lung cancer was 3.6. The patient was functionally fit for standard lobectomy and underwent thoracoscopic right lower lobectomy. During the surgical procedure, the middle lobe vein draining into the inferior lobar vein was clearly visualized (Fig. 2A) but, through the dissection within the major fissure between the middle and lower lobes, another vascular anomaly was recognized: the two main trunks of the inferior lobar vein lied superior to the common basal bronchus. In such instance, to preserve drainage of the middle lobe vein, the two main tributaries of the inferior lobar vein were isolated distally from the anterior aspect and divided using a vascular stapling device, after opening the major fissure, transection of the common basal artery, and identification of the inferior lobar bronchus (Fig. 2B, C). The right lower lobectomy was completed with mediastinal and lobar lymph nodes dissection plus individual transection of the artery supplying the superior segment and the lower lobe bronchus. The patient had an uneventful recovery and was discharged home on the fourth post‐operative day.

## Discussion

Although the middle lobe vein typically enters into the right superior pulmonary vein, some authors have described the veins from the medial and lateral segments of the middle lobe draining directly into the left atrium or into the right inferior lobar vein separately or as a single trunk [4]. During a right lower lobectomy, the confluence of the middle lobe vein and inferior lobar vein must be preserved because a pulmonary venous drainage obstruction from the middle lobe leads to surgical complications as severe lung oedema or extension of the planned lung resection. When such vascular anomaly appears, the inferior lobar vein is identified and dissected distally towards the lung parenchyma to avoid narrowing the drainage of the right middle lobe inadvertently [5]. The inferior lobar vein and its tributaries usually pass behind their respective bronchi and, for this reason, they are more readily dissected from the posterior aspect. In this case, the two main tributaries of the right lower lobe vein lied superior to the common basal bronchus and were carefully dissected from the anterior aspect after opening the anterior portion of the major fissure, transection of the common basal artery, and identification of the lower lobe bronchus. Not all anatomical variants are a common finding on chest CT examination: for this reason, even during VATS surgery, a clear exposure of the anatomical structures is recommended in order to avoid severe operative complications.

### Disclosure Statement

Appropriate written informed consent was obtained for publication of this case report and accompanying images.
